# Neurons in inferior temporal cortex are sensitive to motion trajectory during degraded object recognition

**DOI:** 10.1093/texcom/tgac034

**Published:** 2022-08-18

**Authors:** Diana C Burk, David L Sheinberg

**Affiliations:** Department of Neuroscience, Brown University, Providence, RI 02912, United States; Department of Neuroscience, Brown University, Providence, RI 02912, United States; Carney Institute for Brain Science, Brown University, Providence, RI 02912, United States

**Keywords:** area IT, integration, movement, nonhuman primate, visual perception

## Abstract

Our brains continuously acquire sensory information and make judgments even when visual information is limited. In some circumstances, an ambiguous object can be recognized from how it moves, such as an animal hopping or a plane flying overhead. Yet it remains unclear how movement is processed by brain areas involved in visual object recognition. Here we investigate whether inferior temporal (IT) cortex, an area known for its relevance in visual form processing, has access to motion information during recognition. We developed a matching task that required monkeys to recognize moving shapes with variable levels of shape degradation. Neural recordings in area IT showed that, surprisingly, some IT neurons responded stronger to degraded shapes than clear ones. Furthermore, neurons exhibited motion sensitivity at different times during the presentation of the blurry target. Population decoding analyses showed that motion patterns could be decoded from IT neuron pseudo-populations. Contrary to previous findings, these results suggest that neurons in IT can integrate visual motion and shape information, particularly when shape information is degraded, in a way that has been previously overlooked. Our results highlight the importance of using challenging multifeature recognition tasks to understand the role of area IT in naturalistic visual object recognition.

## Introduction

Objects can be recognized even when visual information is ambiguous. For example, when walking through a foggy park, you might see a blurry shape sitting on the grass in the distance and be unable to figure out what it is. However, if the shape starts hopping, you may perceive it as a rabbit. In this case, the recognizable motion of the rabbit serves as a predictive feature for shape recognition. Here we present an investigation of the neural basis by which predictive motion can be used to recognize degraded visual images.

Past work has shown that stimuli involving motion and shape features, such as structure from motion stimuli (Sáry et al. 1993), can promote crosstalk between dorsal and ventral visual pathways, suggesting that motion and shape might not always be processed in isolation (see Farivar 2009 for a review). As one moves along the ventral visual pathway, neurons exhibit selectivity for more complex features (Gallant et al. 1993; Rust and Dicarlo 2010). In early visual areas, neurons are sensitive to very basic object features, including orientation, size, luminance, contrast, and motion (Hubel and Wiesel 1968; Schiller et al. 1976). Further along the visual pathway, V4 neurons are selective for simple combinations of these features, such as textures, curvature, and color (although the basis of their selectivity is still not well understood) (Touryan and Mazer 2015). Once visual information reaches inferior temporal cortex (area IT), the combinations of features become more complex in a way that is still not completely understood (Zhivago and Arun 2016; Rajalingham and DiCarlo 2019 ; Zhuang et al. 2021). However, it is well known that IT neurons respond to high-order features (Op de Beeck et al. 2001; Tamura and Tanaka 2001) and that this area is implicated in representing categorical information for recognition (Logothetis and Sheinberg 1996; Kriegeskorte et al. 2008; Ritchie et al. 2015). It has been possible to identify 3D features that elicit maximal firing in IT neurons (Yamane et al. 2008) and to extract some computational relationships between features. But the semantic, real-world relationships between features that drive IT neurons remain an active area of research. Furthermore, the superior temporal sulcus (STS), which is anatomically situated between the dorsal and ventral visual pathways, is well known as a site of integration for shape and motion cues (Tanaka et al. 2002; Puce and Perrett 2003; Jastorff et al. 2012). However, much of the work in STS has focused largely on articulated motions (e.g. gestures, walking, motion of limbs), and little is known about the motion patterns of whole objects and their role in object recognition. Although area IT is bidirectionally connected to STS, there has been little evidence of motion processing in area IT, despite recent findings suggesting higher level representations of objects, including the physical properties of objects (Yildirim et al. 2019; Jia et al. 2021).

Moreover, previous behavioral tasks involving motion during object recognition have focused largely on other kinds of motion, including rotation (Vuong and Tarr 2004), structure from motion (Britten et al. 1992; Sáry et al. 1993; Beer et al. 2009), random dot motion (Pilly and Seitz 2009), articulated motion (Jastorff et al. 2006; Singer and Sheinberg 2010; Tyler and Grossman 2011; Schluessel et al. 2015), and complex naturalistic movies (Wang et al. 2012; Russ and Leopold 2015; Haxby et al. 2020; Jääskeläinen et al. 2021). While tasks using these kinds of motions can be informative, it remains largely unknown how trajectory motion plays a role in object recognition, even though trajectory motion is a common component of our perceptual experience. In some cases, motion can provide a strong cue to identification or even define the shape itself (Balas and Sinha 2008; Balas and Sinha 2009; Wang and Zhang 2010; Tian and Grill-Spector 2015). Thus, this experiment was designed to expand our understanding of the role of translational motion in shape processing.

To fully understand vision in natural environments, we must also understand processing of degraded visual information. Common circumstances we might encounter in daily life include recognizing objects in complex scenes among clutter, limited viewing angle, or poor lighting. These are examples of natural shape degradation where a shape is less visible in a way that hinders its recognition. Many methods have been explored to degrade visual stimuli in laboratory tasks, including decreasing contrast (Vogels and Biederman 2002; Namima et al. 2014), partial occlusion (O’Reilly et al. 2013; Tang et al. 2014; Namima and Pasupathy 2021), scrambling objects (Kravitz et al. 2010), morphing objects (Akrami et al. 2009), and salt-and-pepper noise (Emadi and Esteky 2013; Kuboki et al. 2016). Most studies have shown that these methods characteristically reduce the magnitude of neural responses to visual stimuli both in V4 (Kosai et al. 2014), and in area IT (Nielsen et al. 2006; Emadi and Esteky 2013) during visual tasks. A few studies have shown that degradation can have mixed effects on neural responses in prefrontal cortex (Rainer and Miller 2000; Fyall et al. 2017) and that task experience can reduce degradation effects (Rainer and Miller 2000). However, it has recently been shown that some neurons in V4 can respond better to partially occluded shapes (Fyall et al. 2017) and that this is due to feedback from prefrontal cortex to area V4. The same group has also shown that neurons in V4 can respond with selectivity to blurred shapes, suggesting that degraded shapes are not always represented by decreased firing rates (Oleskiw et al. 2018). As area IT has a high degree of interconnectivity with area V4 (Felleman and Van Essen 1991), this suggests that “some” neurons in area IT might also respond better to degraded stimuli, particularly under behaviorally relevant circumstances.

Here we test the idea that reducing shape clarity will cause some neurons in area IT to respond to predictive motion during visual shape recognition. We designed a task that required monkeys to use motion information (2D motion trajectories) to recognize shapes under variable levels of shape degradation. The behavior of the monkeys on this task showed that monkeys can indeed use motion information to recognize degraded shapes. We simultaneously recorded from neurons in area IT while monkeys performed the active recognition task, as well as during passive viewing of the same moving stimuli. We hypothesized that neurons in IT that are selective for shapes would also be selective for their associated predictive motion trajectories. Here we show evidence that IT neurons are selective for both shape and motion trajectories. The design of our experiments allowed us to further reveal that this shape–motion sensitivity is complex: Shape selective neurons are not always selective for their predictive motion patterns, and motion sensitivity evolves over time at the population level. These findings provide evidence for the representation of motion information in area IT during recognition of moving shapes.

## Materials and methods

### Subjects and behavioral sessions

**Fig. 1:**
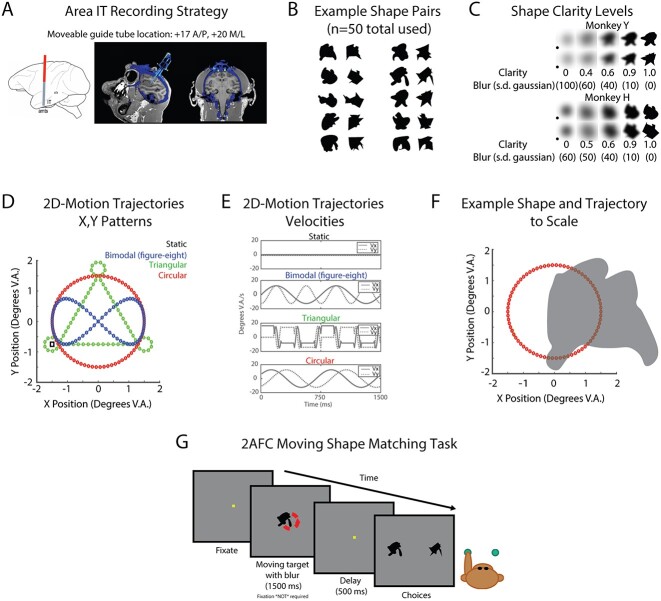
Task design and recording strategy overview. A) Chamber placement and guide tube location for neural recordings from area IT. Left: Sagittal atlas view of approximate recording approach. Right: CT-MRI overlay for Monkey H, sagittal view (left), coronal view (right). B) Example 2D shapes used in behavioral task. Fifty pairs of randomly generated 2D shapes were used in the behavioral task. Each pair, as shown here, consisted of one “blob” (first column) and one “spikey” (2nd column) and was generated from the same randomly overlapping polygons (*n* = 4). Ten example pairs are shown. C) Shape clarity levels used in behavioral task for each monkey. Clarity (Gaussian blur) levels are shown for both Monkey Y (top) and Monkey H (bottom). Shape stimuli were made more ambiguous by using a Gaussian blur with a particular standard deviation (SD) in pixels. Clarity was defined as 1-(blur/100), except in the 0 clarity case. In this condition, for both monkeys, a black circle (see inset) was the underlying stimulus, such that there was no diagnostic shape information that could be extracted from the stimulus. All shape stimuli had transparent backgrounds and were displayed on a gray background during the tasks; edges visible in (C) are an artifact of reproducing the stimuli on a white background. D) The 4 movement patterns used in the task. The *x*,*y* positions of the 4 possible target motion patterns (green = triangular, red = circular, blue = bimodal, black square = static) are plotted. Stimuli started in the bottom left near the overlap of the 3 motion trajectories, except in the static case, where the location is marked with the square. E) Target motion *x*, *y* velocity profiles. These correspond to the motion trajectories in D. F) Example of a shape at 50% opacity with the circular trajectory to scale. Shape is shown at 50% opacity and centered at the rightmost point on the circle trajectory to demonstrate the significant overlap of the shapes when they were moving along the trajectories. G) Example trial of match-to-sample task with moving shapes. The monkey first had to acquire fixation for ~200 ms, after which the fixation spot disappeared, and a moving target appeared for 1,500 ms. The allowable eye position window (not shown) during the target period was −4° V.A. in *x* and *y* to encourage pursuit of the moving target. After 1,500 ms, the target disappeared and the fixation square reappeared for 500 ms for a delay period. At the end of the delay, the 2 static choices appeared and the monkey had 2 s to select an answer using a button press.

Two adult male macaque monkeys were used in this study. In both animals, access to area IT was made possible by the use of a custom ball-and-socket chamber (Schiller and Koerner 1971; Sheinberg and Logothetis 1997) placed over either the right hemisphere (monkey Y) or the left hemisphere (monkey H) at +17 mm anterior, +20 mm lateral (Horsley-Clark stereotactic coordinates). Chamber location was verified using computed tomography (CT) scans for both animals, and the data were processed using a CT-magnetic resonance imaging (MRI) merge in NIH-AFNI software or the Brainsight system (Rogue Research) (see Fig. 1A). All surgeries were performed under isoflurane anesthesia, in accordance with the guidelines published in the National Institutes of Health Guide for the Care and Use of Laboratory Animals and approved by the Brown University Institutional Animal Care and Use Committee.

### Task stimuli

Two-dimensional, randomly generated, geometrical filled shapes were used in the behavioral tasks. The shape set consisted of 50 pairs of shapes, or 100 shapes in total. Half of the shapes were sharp-edged polygons called “spikeys” and their smooth interpolated matches were called “blobs.” The spikey and blob shapes were used to generate a shape discrimination task where the difficulty of discrimination would be controllable and the shapes had the same size, color, and texture. The blobs and spikeys were generated using a previously published algorithm for generating novel shapes (Sigurdardottir et al. 2014). Briefly, the algorithm overlays multiple randomly generated polygons and generates control points derived from the Boolean union of the overlapping shapes. Straight lines between the control points were used to make a novel, closed shape, called the “spikey,” and the edges and corners were smoothed to create the “blob.” Example pairs are shown in Fig. 1B. Shape stimuli were blurred by convolving the shape images with a circular 2D Gaussian kernel with variable standard deviation (SD) (Oleskiw et al. 2018). Five blur levels (i.e. clarity levels) were chosen based on each animal’s behavioral task performance, such that there were 3 levels between the 2 extremes of no blur (100% clarity) and 0% shape clarity (Fig. 1C). Shape clarity was defined as (1 SD of the 2D Gaussian)*100, except in the 0% shape clarity case, because the underlying shape was not a shape from the set. We define blur as the inverse of shape clarity. The underlying shape stimulus of the 0% clarity stimuli was a circle polygon (not the true shape) to ensure that there was absolutely no task-relevant shape information in the motion-only stimulus. Monkey Y had a fainter 0% clarity stimulus than Monkey H to reduce behavioral bias; the values of the SD of the Gaussian used to blur the circle are shown in Fig. 1C. For all pairs, adding blur to the shapes made them more similar to each other in order to make recognizing the shape as either a blob or spikey more difficult.

Three 2D motion trajectories were used in the task. The motion trajectories were designed to be periodic, discriminable, and share a start and end location. They were also designed to have positional overlap and similar average velocities. The trajectories were based on earlier work demonstrating that human subjects could recognize and discriminate between similar periodic, closed-shape motion patterns (Wickelgren and Bingham 2008). The *x*,*y* position patterns of the 3 trajectories (circular trajectory, triangular trajectory, and bimodal [figure eight] trajectory) are shown in Fig. 1D. The periodic trajectories were designed to last 1,500 ms and have a 750 ms period (2 cycles total) to maintain similar average speed. Velocity profiles for the trajectories are shown in Fig. 1E. The trajectories were small relative to the size of the shapes, such that throughout the motion of the shape, there was an overlap at nearly all positions along the trajectories (Fig. 1F).

### Active match-to-sample task

In the 2-alternative forced-choice task, the monkeys were required to report with a button press which of 2 choices best matched a target stimulus. On each trial, the monkey was first required to acquire the fixation spot (~200 ms) within 1° V.A. of the center of the screen, after which a target shape (3° V.A.) appeared for 1,500 ms duration. At target onset, the fixation spot was extinguished and the fixation window was expanded to ±4° V.A. to encourage pursuit of the target. The target either moved in 1 of 3 ways or not at all (static condition). After 1,500 ms, the target was extinguished and the monkey was required to reacquire the fixation spot during the delay. After the delay (500 ms), 2 choices appeared. The monkey then had 2,000 ms to press a right or left button to select which option of the shape pair best matched the target (Fig. 1G). The shapes presented in the choice period were always 100% shape clarity and included the shape that matched the target shape and the corresponding blob or spikey shape. Trials with responses that were too slow (reaction times longer than 2,000 ms) or absent were treated as aborts and were shuffled back in with remaining trials for repetition later within the block. Each shape category was associated with a motion pattern (Monkey Y: blobs-circular, spikey-triangular; Monkey H: blobs-bimodal, spikey-triangular). Both shape categories were associated with the remaining motion pattern and the static condition.

The task conditions were defined by the shape clarity, motion pattern, and shape category (blob or spikey). Trials with no shape information were rewarded randomly on 50% of completed trials, as there was no correct answer. Half of the trials for the session consisted of the single shape pair that was selected for most effectively driving the neuron(s) in that session. The other half of the trials consisted of any shape from the 100 shape set and were included to reduce behavioral bias. Monkey Y completed approximately 5 blocks of 180 trials per session and Monkey H completed approximately 5 blocks of 126 trials per session. During training, monkeys were exposed to the degraded stimuli and difficulty was increased over the course of months by decreasing shape clarity and increasing the number of task conditions. Each monkey was first trained on the match-to-sample task with variable shape clarity and unmoving (static) shapes for 1–2 months. Then, each monkey was trained on the predictive motions (Monkey Y: blobs-circular, spikey-triangular; Monkey H: blobs-bimodal, spikey-triangular) for multiple months before the shared motion pattern was added into the task. The monkeys then each had 2–6 months of experience with all the task conditions interleaved before neural recording sessions began. The behavioral data presented were collected after extensive training on all task conditions. In this dataset, all task conditions were randomly interleaved and counterbalanced for response side; blocks only varied in the number of repetitions for each monkey.

Behavioral performance was calculated as the proportion of correct trials out of the possible number of trials for a given task condition (motion, shape, and clarity). Average reaction times were calculated across all incorrect and correct trials for a given condition. Performance across sessions was calculated by averaging the performance across sessions for a given condition.

### Passive viewing tasks

Passive viewing tasks were used to assess shape selectivity and to select a stimulus pair that would drive neural responses during the active task. The 3 passive viewing tasks had (i) all the 100 shapes with no degradation, (ii) a single pair of shapes (blob, spikey) with multiple shape clarity levels and no motion, and (iii) a shapeless blur moving with each of the motion patterns. During all passive viewing tasks, a fixation spot was shown on the screen and the monkey was required to maintain fixation on this spot for ~250 ms before a stimulus appeared. Multiple stimuli (up to 9) were shown during a single trial, during which the monkey maintained central fixation. Each stimulus was repeated at least 10 times in random order. During passive viewing of the 50 shape pairs without motion, each shape was shown at the center of the screen (size approximately 3° V.A.) for 200 ms with a 100-ms blank interval between stimuli. Online peristimulus time histograms were used to select a stimulus pair for the rest of the session. The shape pair used for a daily recording session was selected based on its effectiveness in driving the neuron recorded that day (or multiple neurons on an array) without any assessment for motion response. The single shape that evoked the largest firing rate from the neuron or majority of neurons was chosen, and its corresponding match was used for the remainder of the session. During passive viewing of the selected single pair across clarity levels, each shape was shown for 200 ms (100 ms between stimuli) with all the clarity levels used during the match-to-sample task. During passive viewing of the moving blur (no shape information), the moving blur with 1 of the 4 motions was shown for the duration of the target in the active task (1,500 ms).

### Eye movement data and analysis

Eye position was sampled by an IR-based camera system (Eyelink) at 1 kHz and moving averages were continuously stored to disk at 200 Hz. At the beginning of each behavioral session, the eye position was calibrated to the computer monitor coordinates by running a standard 9-point calibration procedure (Stampe 1993). Average pursuit trajectories were calculated by averaging the *x* and *y* eye positions during the target period for each task condition.

### Neural data acquisition and analysis

Recordings in inferior temporal cortex (area IT, IT cortex) were conducted with single electrodes or 16-channel V-Probes (Plexon, Inc.). The neural data were amplified and digitized (25 kHz) and then processed in a Tucker-Davis Technologies (TDT) neurophysiology system. The raw data from each channel (electrode) were band-filtered with 2 frequency ranges: local field potential (0.3–300 Hz, downsampled to 1,017 Hz) and single units (300–3,000 Hz). Plexon offline software was used for manual spike sorting. Neurons were excluded from analysis if they were unstable over the course of a session or not driven by any visual stimulus or behavioral task feature and were regarded as task-irrelevant. Task relevant was defined as any significant modulation above pre-stimulus baseline (200 ms before stimulus onset) in 5 task time windows (typical IT visual: 50–200 ms; late visual 200–350 ms; very late visual 800–950 ms; early delay 1,550–1,700 ms; late delay: 1,700–1,850). It was clear that some neurons were not visual in the traditional sense, so we sought to remain agnostic to their role in the task without testing too many time windows (Welch’s *t*-test corrected for multiple comparisons α = 0.008).

To assess sensitivity to shape clarity without motion, we used data from the passive viewing of a single shape pair under multiple levels of clarity. Firing rates were binned into 5 ms bins and responses were assessed from 0 to 300 ms after stimulus onset to account for the possibility of stimulus off responses (passive viewing stimuli lasted 200 ms). We used method previously published to quantify the effect of blur on neurons in V4 (Oleskiw et al. 2018). Briefly, we constructed tuning curves as a function of shape clarity for the blob and spikey stimulus separately. We then calculated 2 metrics from each tuning curve: external clarity factor, which was the shape clarity associated with the largest modulation of firing rate relative to the average activity evoked by the non-blurred, 100% clarity stimulus. The second metric was the modulation index, which was the integral under the tuning curve across shape clarity levels. We then normalized each set of relative responses and conducted principal components analysis. The data from each set of responses (4 sets in total, one for each shape stimulus for both monkeys) were then projected onto the first principal components to produce principal values (PVs) for each stimulus: blob (PV_B_) and spikey (PV_S_). We used the PVs to sort the relative responses to verify the validity of this method in capturing the effect of clarity on neural responses. The PVs were then used to quantify the number of neurons in each population that were excited, inhibited, or had a mixture of responses to variable shape clarity.

Sensitivity to motion was assessed for each neuron using either the early visual period (50–200 ms after stimulus onset) or a 150 ms sliding window (50 ms shift). Motion sensitivity at each clarity level was assessed with a one-way analysis of variance (ANOVA) for each shape class across motion patterns (α = 0.05, corrected for multiple tests) across trials from each of the 3 motion conditions for each shape class (predictive motion, non-predictive motion, and static). A traditional multifactor ANOVA was not tractable, as not all the motion and shape conditions were crossed: the “blobs” and “spikeys” each had a unique motion and only shared one motion pattern and the static condition. To assess motion sensitivity across the entire trial period, each unit was assessed as either being motion or shape selective using a one-way ANOVA using the same correction procedure for multiple tests.

Each session consisted of 4 phases: passive viewing of all the possible task shapes (static, i.e. not moving), passive viewing of a single shape pair across clarity levels (static), the active match-to-sample task, followed by passive viewing of the 0% shape clarity stimulus (4 motion conditions). Eye position, behavioral responses, and neural activity were recorded for all tasks.

### Population decoding analysis

Because neurons were recorded across sessions and not all simultaneously, a pseudo-population of neural responses to representative trials was constructed to represent the data across multiple sessions, serving as input into the decoding analyses. For each condition of interest, a subset of representative trials was randomly selected based on the maximum number of available trials across all sessions. For decoding of the 4 motion conditions, 64 randomly sampled trials (without replacement) were used for each clarity level, except for 0.0 clarity, where randomly sampled 96 trials were used for decoding. For each 150 ms time window, neural firing rates were concatenated as features for decoding each trial (no. of features = no. of neural responses in the pseudo-population). The process was repeated for multiple iterations to capture more of the data in the decoding analysis and to reduce variability in decoding accuracy due to sampling bias.

Support vector machines (SVMs) were trained with a linear kernel using MATLAB. Binary learners with the “one-vs-one” approach were used, meaning that for each binary learner, one class was labeled as the positive class and another was labeled as the negative class; others were ignored. Cross-validation within each step was conducted using leave-one-out training and testing of each trial repeatedly, across all trials in the single decoding analysis. Accuracy was computed using the error of the cross-validated result or the fraction of trials labeled correctly by the decoder. Variance of the decoding accuracy was computed and averaged across iterations (over re-selection of trials for each neuron) to get the standard error of the mean decoding accuracy. A separate model was fit for each 150 ms time window (50 ms sliding window), following previous work demonstrating success in using these timing parameters for decoding neural responses in area IT (Babolhavaeji et al. 2014).

Shuffled label tests were performed to establish decoding performance at chance level to capture bias in the data. Previous work has shown that comparing performance to only theoretical chance level can lead to misinterpretation of decoder performance, as an increase in sample variance of data values will also increase the chance of rejecting the null hypothesis when compared against theoretical chance (Combrisson and Jerbi 2015). Thus, following other recent developments in decoding toolboxes (Bode et al. 2019), repeats of all original analyses using the original data (i.e. randomized trial selection, no. of iterations of a *k*-fold cross-validation) were conducted using a random assignment of labels to exemplars to produce the “shuffled labels” level of chance. This accounted for any potential biases in the original data that might affect true chance level accuracy and would not be accounted for using theoretical chance levels (although both are shown in each decoding figure). The original and shuffled label analyses were otherwise identical.

## Results

We designed a task and recording strategy to explore the relationship between shape ambiguity and motion trajectory in area IT, an area known for its involvement in visual form processing (see Conway 2018 for a review). We hypothesized that when shape information is degraded, IT neurons responding to shape information would respond more to movement during recognition. Based on behavioral data, we show that the animals could recognize the motion trajectories and use them to select the correct shape even when shape was degraded. Analysis of IT neural responses during the task shows that firing rates did not always decrease with shape degradation and that neurons exhibited multiple kinds of responses to shape clarity. Moreover, neurons exhibited a variety of responses to stimulus motion in time. Analysis of motion sensitivity across the target and delay period and population decoding suggests that a significant proportion of neurons responded to motion throughout the target period. Lastly, we compared decoding performance from the active task (where smooth pursuit was allowed) to decoding performance from the passive task (fixation required), to demonstrate that decoding performance was not solely dependent on eye movements.

### Monkeys can learn to use motion to recognize degraded shapes

**Fig. 2:**
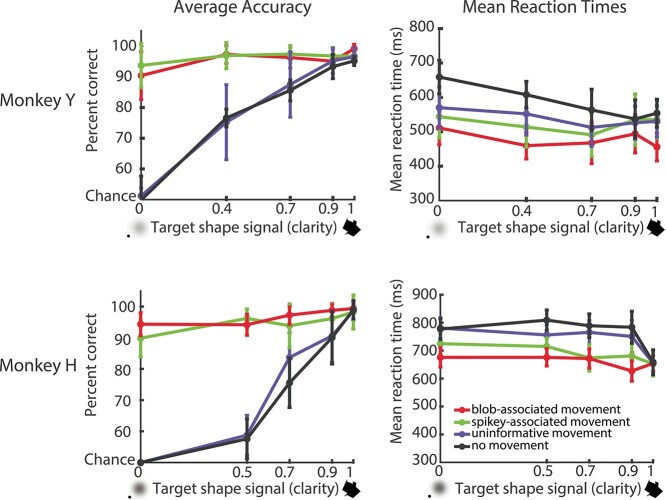
Behavioral performance and average reaction times during active task. Overall behavioral performance across sessions included in neural data analysis for both Monkey Y (28 sessions) and Monkey H (33 sessions). Average performance for each monkey (left) across the 4 motion conditions demonstrates the use of the motion–shape associations in low shape signal (clarity), as performance stayed near ceiling for the hardest clarity levels when the target was moving with the 2 predictive motions. Performance decreased to chance for the most difficult clarity level (target shape signal 0) for the uninformative motion and static conditions. Reaction times were slightly slower for the static condition for both monkeys (right, black curves). Error bars SEM across sessions.

Two monkeys completed approximately 30 sessions of the match to sample task (33 sessions for Monkey H, 28 sessions for Monkey Y), which required matching a shape to a target that varied in motion and shape clarity. The easier shape clarity trials could be completed correctly using motion or shape information, whereas the difficult clarity levels, and particularly the condition with no shape information (0.0 clarity), required the use of motion–shape associative knowledge. The motion trajectory mappings were different for the 2 monkeys, so the particular motion–shape associations were not critical as multiple mappings were learnable. As shape clarity increased, accuracy increased from chance level to around 90% for the (non-predictive) motion conditions that both shape categories had in common (static and shared motion) (Fig. 2, left). However, for the motion patterns uniquely associated with only one shape class (blob or spikey), performance was not affected by shape clarity and remained around 90% accurate across all shape clarity levels. This demonstrated that the monkeys were using the learned motion–shape associations to select correct responses, even when shape information was degraded or unavailable. Reaction times varied slightly across the motion conditions despite the 500 ms delay before choice period. Reaction times trended slightly faster (although not statistically different across every clarity level) for both monkeys in the conditions that involved the use of the motion–shape associations (red & green curves, Fig. 2, right).

The goal was to create a behavioral task for monkeys to recognize and select shapes based on motion patterns. The behavioral data demonstrate that the monkeys can flexibly use motion information when shape information is not available to select the corresponding shapes. Both monkeys learned the motion patterns associated with each shape class to properly choose a shape that matched a degraded moving shape. We next asked how neural responses in area IT are affected by shape degradation and motion.

### IT neuron responses to shapes are modulated by shape clarity

**Fig. 3:**
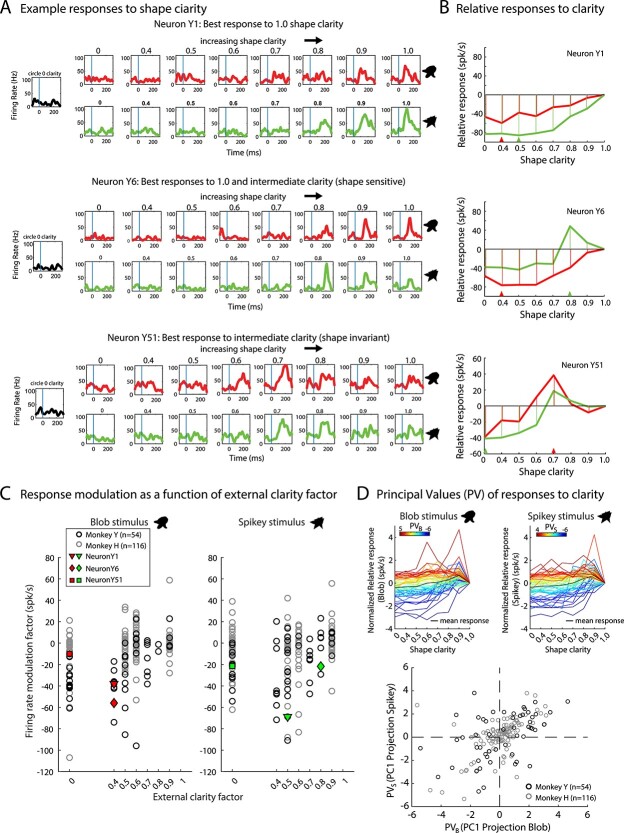
Model-free analysis of sensitivity to shape clarity during passive viewing. A) IT neurons can respond differently to varying shape clarity. Top: An example of a neuron (Y1) whose firing rate increases with increasing shape clarity for both shapes in a pair. Average firing rates for each shape (blob, red; spikey, green) at clarity levels ranging from 0 to 1.0 are shown. Middle: An example of a neuron (Y6) with a mixture of the kinds of responses seen in (A) and (B), that responds best to the blob with 1.0 clarity and spikey with 0.8 clarity. Bottom: An example of a neuron (Y51) that responds best to 0.7 clarity for both shapes. B) Relative firing rate responses as a function of shape clarity for the 3 example neurons in (A). Response modulation was determined by calculating the integral of the relative responses across clarity levels (hatching). An external clarity factor was defined as the magnitude of shape clarity that evoked the largest modulation in firing rate with respect to the response to the 100% clarity stimulus. An external clarity factor was calculated for each shape stimulus: blob (red triangle) and spikey (green triangle). C) Firing rate modulation plotted as a function of external clarity factor for each shape stimulus. Example neurons in (A) and (B) are highlighted. D) Quantification of sensitivity to shape clarity using the PVs. Top: Normalized relative responses for Monkey Y colored by the PV, calculated from the first PC of these normalized responses, for each shape stimulus. More positive values (red) indicate that neurons responded with higher firing rates to intermediate or low levels of shape clarity than shapes with no blur. More negative values (blue) indicate that those neurons exhibited decreased firing rates with decreasing shape clarity. Mean normalized responses are plotted in black (see Supplementary Fig. 1 for Monkey H). Bottom: Scatter plot of all PV values (*x*-axis: PV for blob, PV_B_ and *y*-axis: PV for spikey, PV_S_) for Monkey H and Monkey Y.

During each behavioral session, activity from one or more neurons from area IT was recorded using single electrodes or multi-contact probes. Neurons were first assessed for responsiveness to each of the possible task shapes using passive viewing (*n* = 100 shapes). The shape that elicited the highest firing rate (i.e. single blob or spikey) was selected from the set and this shape, along with its counterpart (blob or spikey), and these were used for the remainder of the session. Neurons were then assessed for sensitivity to shape clarity using the selected blob and spikey pair. The 2 shapes in the pair were shown with variable levels of shape clarity in a passive viewing paradigm. The pair was shown using at least the same clarity levels as those used in the active task. We had hypothesized that as shape clarity increased, firing rates would increase. Although some neurons exhibited increased firing rates with increasing shape clarity for both shapes (Fig. 3A, top), we also observed 2 other kinds of responses: increased firing rate with shape clarity for one shape and not the other (Fig. 3A, middle), or maximum firing rate with intermediate shape clarity (Fig. 3A, bottom). In order to characterize the effect of shape clarity across the population, we performed a previously published analysis of blur modulation used to characterize the effect of blur on neural responses in V4 (Oleskiw et al. 2018). For each neuron, we constructed tuning curves as a function of shape clarity for the blob stimulus and spikey stimulus (Fig. 3B). We then calculated 2 metrics from each tuning curve. The first metric was the external clarity factor (red and green triangles, Fig. 3B), which was the shape clarity associated with the maximum modulation of firing rate relative to the average activity evoked by the non-blurred, 100% clarity stimulus. The second metric was the modulation index, which was the integral under the tuning curve across shape clarity levels for each stimulus (Fig. 3B, hatching). Figure 3C depicts the modulation factor as a function of external clarity factor for all neurons in both pseudo-populations. In this space, there is a continuum of responses and it is visible from the scatter plot that there are neurons with positive and negative firing rate modulation by shape clarity, and a range of external clarity factors that evoked the largest modulation in firing rate. The 2 metrics for the single neuron examples from Fig. 3A and B are highlighted in Fig. 3C, demonstrating that neurons could exhibit different external clarity factors depending on the stimulus (blob or spikey). We took this analysis one step further in order to summarize and quantify the neural responses to clarity and shape stimulus. We conducted dimensionality reduction on the normalized relative responses and computed the projection of the data on the first principal component (PC1) to produce 2 PVs, a principal value for the blob stimulus (PV_B_) and a principal value for the spikey stimulus (PV_S_). A positive PV indicated that a neuron’s firing rate was modulated by intermediate shape clarity with respect to the 100% clarity stimulus. A negative PV indicated that a neuron’s firing rate was reduced by low shape clarity. Overlays of the normalized, relative response curves for all neurons, colored by the PV, showed that the PV was a reasonable metric for quantifying modulation by blur (Fig. 3D, top and Supplementary Fig. 1). We then used the PV_B_ and PV_S_ to quantify the number of neurons in 4 categories: (A) PV_B_, PV_S_ > 0 firing rate enhanced; (B) PV_B_, PV_S_ < 0 firing rate suppressed; (C) PV_B_ > 0, PV_S_ < 0 firing rate enhanced for blob and suppressed for spikey, and (D) PV_B_ < 0, PV_S_ > 0 firing rate suppressed for blob and enhanced for spikey. For Monkey Y, we found the largest proportion of neurons (*n* = 23, ≈ 46%), had firing rates that were enhanced by decreased shape clarity. The next largest subpopulation had reduced firing rates with lower shape clarity (*n* = 18, ≈33%). In the categories that quantified mixed sensitivity based on shape identity and clarity, we found lower proportions of units (*n* = 5, ≈ 9% and *n* = 8, ≈15%, respectively). For Monkey H, we found similar trends across these 4 categories (*n* = 53, ≈46%; *n* = 31, ≈27%; *n* = 15, ≈13%; *n* = 17, ≈ 15%),

Given the observed variation of single-unit responses to shape clarity, it was evident that traditional metrics of shape selectivity would not provide an accurate or useful representation of a neuron’s responsiveness to shape information. For example, neurons could sometimes exhibit their highest firing rate at 1.0 clarity for a blob, but an even higher firing rate for the corresponding spikey at lower clarity (Fig. 3A, middle and Fig. 3A, middle). This type of response was not consistent across all shape pairs for this neuron, or for others, and thus not tractable with traditional metrics, although they were calculated for all neurons. Previously reported selectivity metrics, including degree of selectivity, breadth, and broadness (Mruczek and Sheinberg 2012), were calculated for all neurons, but no significant groupings or clusters were found, and there was no apparent relationship between the metrics and the 3 main kinds of responses observed. Thus, we chose to shift our approach to investigate how motion sensitivity, rather than selectivity, might change with shape clarity.

### IT neurons show clarity-dependent sensitivity to motion

**Fig. 4:**
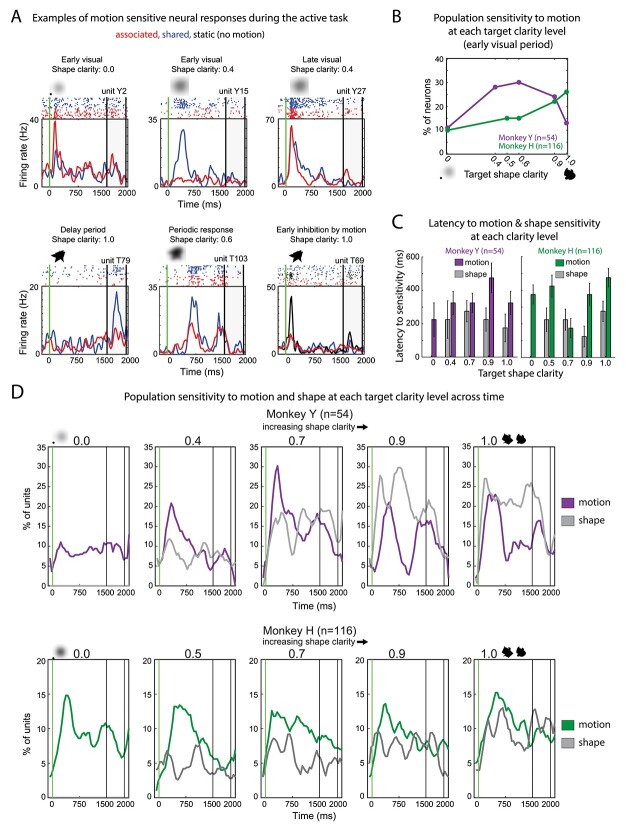
Multiple kinds of neural responses to motion and population sensitivity to motion across time. A) Single unit examples of motion sensitivity. In each subplot, the response to the same shape stimulus (inset) to 2 different motion patterns (associated with that shape and not) is shown, revealing a variety of differences between motion conditions for the same shape. B) Quantification of the proportion of motion sensitive units during the early visual period. The plot is of the proportion of units that were motion sensitive at each target clarity, only during the early visual period (50–200 ms after stimulus onset). C) Latency to shape and motion sensitivity for both monkeys for units that were motion and/or shape sensitive. Left: Bar plot of shape and motion latency across all clarity levels for Monkey Y. Right: Analogous bar plot for Monkey H. Error bars SEM. D) Population motion and shape sensitivity across the entire target and delay period for each clarity level. In each subplot, the motion sensitivity (150 ms window, 50 ms sliding window) is shown across time using the same procedure used to generate (B) for a single time window. Percentage of units sensitive to shape for each clarity level is plotted in gray and sensitivity to motion is plotted in purple (monkey Y, top) or green (monkey H, bottom).

We asked whether neurons were motion sensitive and how motion sensitivity changed with target shape clarity. As neurons were not assessed for both shape clarity and motion with passive viewing, data from the active task were used for this analysis. To assess for motion sensitivity, firing rates from each motion condition for a particular shape stimulus (blob or spikey at a particular clarity) were compared using a one-way ANOVA (significance level *P* < 0.05 then Bonferroni corrected for multiple comparisons). Thus, the main comparison was the neural response to a blob or spikey moving with the predictive movement pattern, the shared movement pattern, or no movement at all. The neurons exhibited a variety of responses to the motion conditions at various clarity levels (Fig. 4A). Surprisingly, there were multiple types of responses to motion of the same shape stimulus. Notably, motion sensitivity varied in time and occurred at a variety of clarity levels. The first subplot shows the neural responses (for unit Y2) to the predictive motion and shared motion at 0% clarity. In this example, the predictive motion evokes a significantly larger early visual response than the shared motion pattern for the same stimulus that has no relevant shape information. In contrast, the second subpanel shows an example of a unit for which the shared motion pattern evoked a much larger early visual response than the predictive motion pattern for a significantly blurred stimulus. Across this series of examples, the timing of and magnitude of peak activities in the single-unit responses are modulated by motion type (shown by the colors in Fig. 4A). We observed, following our analysis of sensitivity to shape clarity, that neurons that were motion sensitive at one clarity level were not necessarily motion sensitive at all clarity levels. Thus, we assessed the proportion of neurons that were sensitive to motion at each clarity level, starting with the early visual period of 50–200 ms after target onset (Fig. 4B). Monkey Y had a larger proportion of units that were motion sensitive at intermediate clarity levels (but fewer units overall). There was a slight increase in motion sensitivity for both monkeys as clarity increased but that was not statistically significant. Despite the differences between monkeys, these data show that IT neurons can be sensitive to motion at different clarity levels. We next investigated motion sensitivity beyond the early visual period.

### Neural responses show dynamic motion sensitivity

The analysis above focused on the initial visual period, which is the time of typical visual responses in area IT. Previous work has suggested that degraded stimuli (such as the blurred ones used in this task) promote recurrent processing that might occur beyond the initial visual period (Wyatte et al. 2012; Tang et al. 2018; Namima and Pasupathy 2021; Ernst et al. 2021, Apr 21). Furthermore, the trajectory period was extended in time (1,500 ms); thus, it was natural to consider sensitivity to motion throughout the target period. We hypothesized that because motion sensitivity requires integration over time, that the latency to shape sensitivity might vary from that of the latency to motion sensitivity. To assess sensitivity to motion and shape information during the target period and delay, we used a sliding window analysis and sequential assessment of sensitivity to motion and shape class. Each unit was assessed as either being motion or shape selective (*P* < 0.05, Bonferroni correction for multiple tests), representing significant modulation by either motion trajectory or shape class. For each neuron that was assessed as motion sensitive and/or shape sensitive, we calculated the time to this initial sensitivity (Fig. 4C). We found that latency to motion sensitivity was longer than shape sensitivity although not statistically significant across both monkeys for all clarity levels. Due to the heterogeneity of motion sensitive responses observed at the single neuron level (Fig. 4A), we hypothesized that sensitivity to motion and shape information might fluctuate in time and that the participation of units across the trial period might also fluctuate. Figure 4D shows motion sensitivity (purple or green) and shape sensitivity (gray) of the population across the entire target and delay period at each clarity level, for both monkeys. Critically, these are not metrics for visual responsiveness, but rather significant sensitivity to the visual features of the stimulus, i.e. motion of the stimulus (shared motion vs. static vs. blob-predictive vs. spike-predictive) and shape (blob vs. spikey). This analysis suggested that sensitivity to motion fluctuates in time and that the population could potentially represent motion patterns and support discrimination between motion conditions, even with degraded shape information.

### Population decoding shows decodable representation of motion information

**Fig. 5:**
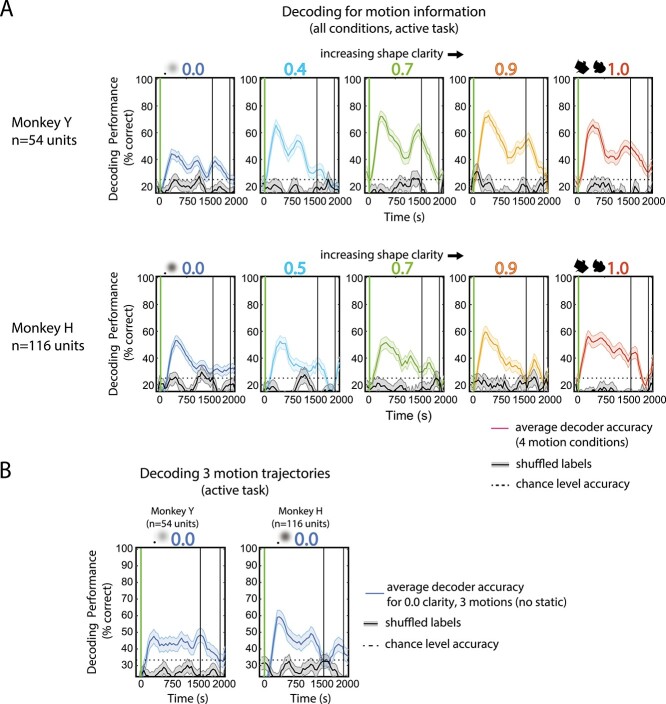
Decoding motion conditions from neural population activity. A) Decoding for motion condition across clarity levels. Multiclass (4-way) decoding of motion condition (static, shared, blob-predictive, spikey-predictive) was done using 150 ms windows, 50 ms sliding window; a separate model was used for each time window. Each panel is the average decoding performance, or average percent of correctly labeled trials, across iterations (of random resampling across trials and sessions) to decode 64 trials of combined neural activity across the ~30 recording sessions. 0.0 clarity panels (far left) used 96 trials for decoding. Error bars SEM. Trial labels were shuffled and decoding analysis was repeated with the same parameters and numbers of iterations to create statistical chance level accuracy. Top: Average decoding performance for clarity levels 0.0 through 1.0 for Monkey Y. Bottom: Monkey H. B) Decoding for motion trajectory without static condition at 0.0 clarity. Multiclass (3-way) decoding of motion condition (shared, blob-associated, spikey-associated) was done using the same procedure as in (A), except using 72 trials for decoding. Error bars are SEM across iterations of decoding.

Motion sensitivity analyses showed that different units were responsive to motion at different levels of clarity, but it remained unclear whether the pseudo-populations could discriminate the motion trajectories throughout the target or delay period. To determine if motion could be decoded throughout the trial, population decoding was carried out for the 4 motion conditions (static, shared, blob-predictive, spikey-predictive) for all clarity levels using SVMs (see Section 2.7). Figure 5A shows the average decoding performance for decoding 4 motion patterns for both monkeys for each clarity level. The decoding performance curves have a generally double-peaked shape in time, cycling around the time of the 2 cycles of motion (with some blurring from the smoothing and window size). Decoding accuracy is lower for 0.0 clarity yet above both theoretical chance and shuffled label decoding performance. To exclude the possibility that the decoders were mostly extracting information related to motion versus static conditions, we also performed decoding on only the 3 movement trajectories and found that decoding performance remained above both theoretical and shuffled chance levels (Fig. 5B). As there is no object-related information present in these trials, the only way a decoder could extract information would be from motion information related to neural activity. Thus, these decoding analyses demonstrate that information about motion condition could be decoded from the pseudo-populations of area IT neurons. Because the monkeys’ eye movements were not constrained during the target presentation phase (i.e. they were allowed to pursue the shapes as they moved along the motion trajectories), we investigated if the decoding performance could be attributed to changes in eye position as opposed to object motion.

### Pursuit eye movements do not account for accuracy of motion decoding

**Fig. 6:**
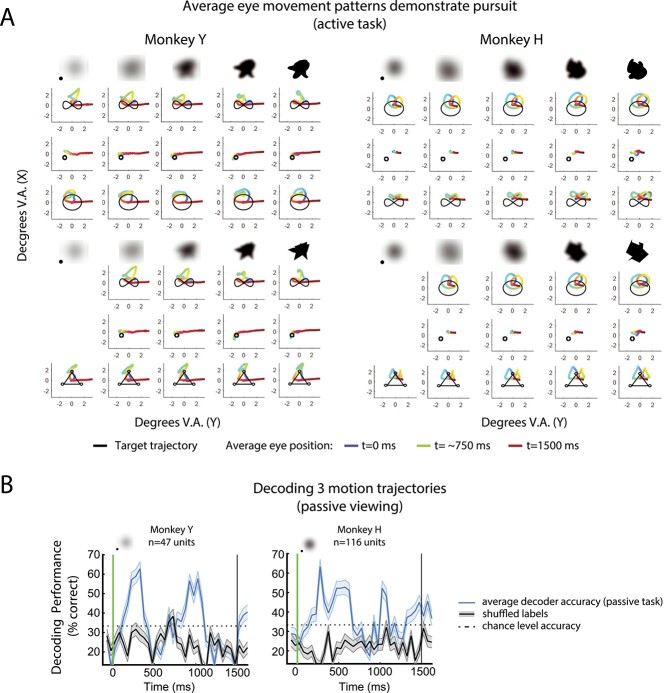
Eye movement data and decoding of motion-only conditions in active and passive tasks. A) Eye movement data demonstrate that both monkeys pursued the moving targets. Average eye positions during the target period (1,500 ms, color) are overlaid with the actual shape trajectories (black) across all shape + motion + clarity task conditions. Each panel is a single motion and shape clarity condition; shape clarity increases from left to right across columns. At the top of the column the shape (blob or spikey) is shown; shapes not to scale and square edges were not visible during the actual tasks. Top rows show average eye movements for blob shapes across the 3 motion conditions (bimodal, static, predictive motion). Bottom rows show average eye movements for spikey shapes across all 3 motion conditions (bimodal, static, predictive motion). Average eye position is color coded from early to late (blue, green, red). Left: Monkey Y, right: Monkey H. Averages are across all correct trials and behavioral sessions. B) Multi-class (3-way) decoding of motion condition during the passive viewing task (shared, blob-predictive, spikey-predictive) at 0.0 clarity for both monkeys, using 36 trials for decoding. Decoding performance is shown as the average percentage of correctly labeled trials across iterations. Error bars are SEM across iterations.

During naturalistic object recognition, eyes naturally follow a moving object of interest. To allow for more naturalistic recognition in this matching task, the monkeys were not required to fixate. Rather, they were allowed to pursue the moving targets but had to maintain their eye position within a large fixation window that was ±4° V.A. of the center of the screen. Eye movement data demonstrated that both monkeys pursued the targets, and a transform of the original trajectory patterns was visible in the average eye movement patterns (Fig. 6A*)*. Average eye position data show anticipatory rightward saccades near the end of the target period (red line rightward) for both monkeys, reflecting a bias to acquire the right choice object first during the choice period. Both monkeys show some drift in eye position during the static conditions. Overall, the eye movement patterns were stereotyped and did appear to cover a smaller spatial range as clarity decreased (i.e. smaller eye movements were made). The eye movement data show that although the motion trajectory was the same across clarity levels, the eye movements in response to the motion and clarity changed across conditions.

Because the monkeys were pursuing the moving stimuli but not the static stimuli, it seemed possible that the population decoders were extracting information related to whether the monkeys were moving their eyes throughout the target period. However, as previously shown in Fig. 5B, excluding the static condition from decoding analyses did not reduce decoding accuracy to below shuffled chance or theoretical chance levels. To further investigate the role of eye movements on the population decoding results, we performed decoding of the same 3 motion patterns during passive viewing, when the monkeys were required to maintain fixation (Fig. 6B). Decoding of neural data during passive viewing was also significantly greater than shuffled and theoretical chance, supporting the idea that eye movements were not the sole contributor to decoding of motion condition in previously shown decoding analyses. Interestingly, decoding of data from the passive task showed a much more distinct bimodal shape than decoding of the neural data from the active task. This suggests that the decoders were likely relying on population activity related to features other than eye movements. However, it is possible that the small differences in retinal position of the stimulus across the conditions (perhaps due to quality of pursuit) could contribute to the decoding performance of motion patterns. This would imply that these IT neurons would have very refined spatial selectivity, which was not otherwise apparent in the data.

## Discussion

Our central finding is that neurons in area IT are sensitive to 2D trajectory motion in a task context where shape information is degraded or absent and motion can be informative. Behavioral data demonstrated that when shape information was degraded, but motion was informative to shape identity, the monkeys could use this information to choose the shape that best matched the target stimulus. Analysis of neuronal firing rates and sensitivity to task features, namely shape and motion information, demonstrated that individual neuron responses to shape clarity and motion fluctuate in time. Our ability to decode motion trajectory using the neural data indicates that information about motion was present in the population responses. Eye movements were permitted during the task to make the recognition of moving objects more naturalistic. Allowing pursuit does add an additional challenge in our interpretation of the decoding results. However, decoding motion from the passive viewing of moving, blurred shapes, showed that eye movements were not solely responsible for the decoding of the active task neural data. These analyses suggest that neurons in area IT have access to motion information in the context of degraded shape recognition.

The goal of the study was to investigate motion processing in area IT during recognition of moving objects. We began this study with the hypothesis that shape and motion are easily and naturally combined as visual features, and that because IT cortex plays such a critical role in object recognition (see Conway 2018 for a review), it could have access to motion information under certain conditions. Specifically, we hypothesized that when shape information is degraded, informative motion information might support object recognition, and that neurons in IT cortex might be driven by motion information to compensate for a lack of shape information. As outlined in Section 1, there are multiple reasons why one might think IT neurons could play a role in the processing of motion during visual object recognition: the demonstration that motion can afford shape recognition (Marr and Nishihara 1978; Lawson et al. 1994; Mitsumatsu and Yokosawa 2003; Vuong and Tarr 2004; Friedman et al. 2009; Pike et al. 2010; Setti and Newell 2010; Nankoo et al. 2017), the role of IT cortex in coding temporal and structural information about visual associations (Sakai and Miyashita 1991; Morita and Suemitsu 2002; Eifuku et al. 2010), sequences (Meyer and Olson 2011; Kumar et al. 2017; Ramachandran et al. 2017; Kaposvari et al. 2018) and action patterns (Singer and Sheinberg 2010; Vangeneugden et al. 2011), and neural modulation based on behavioral experience (Li et al.^1993^; Grill-Spector et al. 2006; Mruczek and Sheinberg 2007; Li and DiCarlo 2008). Furthermore, past work has also shown that prefrontal cortex carries motion and direction information (Zaksas and Pasternak 2006; Hussar and Pasternak 2009) and sends task-relevant feedback to IT cortex (Tomita et al. 1999; Kar et al. 2019; Kar and DiCarlo 2021). Given the high level of interconnectivity between visual areas, and feedback projections from frontal areas to visual areas, prior work would suggest that neurons in area IT could possibly access motion information to support object recognition in the face of shape uncertainty.

Reports of IT neurons responding better to degraded images than crisp ones are limited (Namima and Pasupathy 2021), but it is not surprising that degraded images could be coded in this way, as somehow the brain does achieve recognition under various kinds of visual ambiguity (Wyatte et al. 2012; Emadi and Esteky 2013). Most studies of area IT neurons use rapid presentation of clear images in initial surveys for neurons, select cells based on image sensitivity and selectivity using single electrodes, and average across neurons to analyze population responses, all of which could obscure the properties of individual responses (and averaging did obscure individual response characteristics in our data and population-level averages showed no significant differences between task conditions). With the adoption of high channel count recording probes (e.g. NeuroPixel and others), inspection of individual units in real time becomes more and more challenging. This not only necessitates a different kind of neuronal sampling but also makes it more likely that one might observe unexpected ranges of selectivity (such as preferred responses for blurred stimuli). Perhaps most neurons in IT cortex respond to clear images, and those that respond better to blurry shapes are sparse. Our data do not support this claim, as a nearly even number of neurons were found having selectivity for blurry shapes as crisp ones, and there appeared to be a gradient of responses (not just 2 distinct classes). Furthermore, the different kinds of responses could be observed within 100 μm of each other on the multichannel probe, suggesting that neurons with differing response properties are highly intermingled and possibly interconnected. Future work could investigate the properties of these neurons that appear selective for degraded images. This would require a slightly different approach than most single-unit recording studies in IT cortex.

Recent work has shown tuning for blur and occlusion in V4 (Fyall et al. 2017; Oleskiw et al. 2018), which send direct projections to area IT. It would be interesting and useful to see how neurons in V4 might be transmitting information about stimulus ambiguity to neurons in area IT. Furthermore, this raises the question of whether the motion sensitivity we observed is true motion sensitivity or “degraded feature” sensitivity. Here we only combined motion with shape, but it is easy to imagine an extension of the task employed here where multiple secondary features (e.g. motion, color, and texture) were bound with shape in a recognition task to determine if there are neurons that support recognition of degraded shapes across all these different feature dimensions (e.g. true multidimensional feature integrators) or whether there are distinct subpopulations responding to degraded features that support shape recognition and thus object recognition. Such an experiment could also compare different methods of degradation, which might also affect how IT neurons respond to degraded shape stimuli. Perhaps blur is a special kind of degradation of shape, which motion can induce (i.e. motion makes shapes blurry) and thus promotes the interaction we observed in our data at the level of neural responses. The Gaussian blur used in this study is different than ocular motion blur, which could be induced by making the shape move in a way that makes it blurred, thereby disrupting object recognition. More work needs to be done to disentangle motion and blur interactions in area IT, but this study is the first to demonstrate that these interactions exist.

Decoding of neural population activity was also used to demonstrate that neurons in IT cortex could discriminate between motion conditions, even when shape information was not available. We interpret this as a representation of motion. One question that arose from this result was what was being decoded? The motion conditions were decodable in both passive and active tasks, with and without fixation required, suggesting that eye movements were not leading to the decoding results observed. Past work has suggested that small differences in stimulus position are not sufficient to drive differences in neuronal responses in area IT during free-viewing (DiCarlo and Maunsell 2000). In these experiments, the researchers trained monkeys to identify shapes in fixating and free-viewing conditions and designed the experiments such that retinal stimulation was “nominally identical” but did not eliminate small changes in stimulus position that might evoke firing rate changes in early visual areas. Critically, they found that over 90% of neurons in IT had statistically indistinguishable responses when comparing free-viewing versus fixated viewing of the small shapes. Our shape stimuli had extensive overlap along the trajectories and moved in a range of approximately ±2° V.A., which is significantly smaller than the average receptive field size of an IT neuron on a plain background without distractors (Ito et al. 1995; Rolls et al. 2003). Thus, it is unlikely that small changes in stimulus position due to retinal slip during pursuit account for our ability to decode motion from the pseudo-populations, which were also accumulated over many different shape stimuli used across sessions. Furthermore, analysis of motion sensitivity showed the heterogeneity of responses neurons could have to the motion conditions, suggesting that trajectory representation is happening at a population level, not in single neurons. As motion trajectories are positional sequences, future work could investigate the distinction between motions that are predictive and non-predictive and leverage previous work on the responses of neurons in area IT to sequential information (Meyer and Olson 2011; Kumar et al. 2017; Ramachandran et al. 2017; Kaposvari et al. 2018). Simultaneous recordings from large groups of IT neurons would also enable analyses to determine how trajectories might be coded within subpopulations of IT neurons; the work presented here was limited to using pseudo-populations as a proxy for widespread joint population signals. Large scale recordings could shed more light on what was truly being decoded, whether it was indeed any motion information, or motion information that had been behaviorally linked to shapes (or shape categories).

An additional limitation of our study was that we were unable to assess selectivity for the motion patterns that predicted each shape class. To address whether neurons preferring clear blob stimuli over spikey stimuli also prefer the blob-predictive motion, one would need a full cross of the motions and shapes to show selectivity for the shape predictive motion independent of the shape (i.e. spikey moving with blob-predictive motion). While this variation of the experiment seems ideal, we experienced considerable difficulty in training the animals to learn the motion shape associations and were concerned that crossing the motions and shapes would interfere with task performance. Thus, we did not cross the predictive motions and shapes in this experiment. Future work could expand on the number of motion trajectories, make the shape stimuli more heterogeneous, and explore the potential of completely crossing all motions and shapes to explore both shape and motion selectivity. In our experiment, we interpreted differences in responses to the same shape moving differently as motion sensitivity. Future experiments could investigate selectivity for motion in area IT.

If area IT has access to motion information, as our data suggest, how is this related to general motion responsiveness elsewhere in the brain? We cannot discriminate between the possibilities of motion and shape integration and motion acting as a cue with the data from this study. Any motion representation in area IT might support recognition, but more experiments would be required to clarify the role of motion sensitivity in area IT during recognition of moving objects. A first step toward understanding the representation of motion information during moving object recognition might be a direct comparison with neural responses in areas of STS that respond to biological motion and point light displays. STS is well known for containing neurons that exhibit selectivity for motion, form, and conjunction of these features (Saito et al. 1986; Oram and Perrett 1996; Jellema et al. 2004; Schultz et al. 2005; Hein and Knight 2008; Jastorff et al. 2012). Recording in STS and area IT simultaneously could provide a reference for gaining a better understanding of what was being decoded from neurons in IT cortex, as well as the different roles that area IT and STS play in moving object recognition.

The data from this experiment cannot be used to discern whether the neurons that respond to motion information when shape is degraded are only motion sensitive, or if they are sensitive to other secondary features and context that might support object recognition when shape information is weak or unavailable. Although such responses have not been commonly reported, it is not surprising that some neurons in area IT respond better to degraded images than crisp ones, as the brain must be capable of somehow recognizing objects under degraded conditions, with limited information, and it is well understood that area IT plays a critical role in object recognition (Conway 2018). A recent study demonstrated that neurons in area IT indeed play a role in recognition of degraded patterns, and although onset latencies were not affected by the level of noise, neurons could be characterized as accumulating evidence (Kuboki et al. 2016), which implies that these neurons must integrate information over time, a critical feature required for coding of motion information. Furthermore, recent work has also demonstrated that neurons in area IT can also encode category-orthogonal properties, including those that are often considered lower-level features, when behaviorally relevant (Hong et al. 2016). Our data suggest that motion is represented by different neurons depending on shape clarity, which suggests the possibility that neurons in area IT can flexibly integrate shape and non-shape features to afford recognition, but that some neurons have a propensity to encode these secondary features more than others. Thus, we hypothesize that the neurons we found to be motion sensitive are likely to be sensitive to other features as well, but perhaps play a larger role in population responses in area IT when the primary object feature processed by this brain area, shape, is degraded or unavailable. This interpretation raises the question of whether such response properties (i.e. responding to features other than those traditionally coded in area IT) associated through task training are present in this region before any learning has taken place? It might be possible to determine this using a task requiring the monkeys to learn to recognize new degraded objects while recording from the same neurons during learning.

In summary, the data here suggest that neurons in IT cortex have access to motion information under certain task conditions. Furthermore, the data show an interaction between sensitivity to motion and sensitivity to shape clarity at the single neuron level, which suggests that motion sensitivity in area IT might have been overlooked due to the use of paradigms using rapid presentation of crisp visual stimuli. The paradigm and data here provide a useful platform for future investigation of motion and ambiguity in area IT, both of which are crucial to a clear understanding of object recognition more generally.

## Supplementary Material

SupplementalFigure1_tgac034
